# How Do Family Court Judges Theorize about Parental Alienation? A Qualitative Exploration of the Territory

**DOI:** 10.3390/ijerph19137555

**Published:** 2022-06-21

**Authors:** Telma M. Marques, Isabel Narciso, Luana C. Ferreira

**Affiliations:** CICPSI, Faculty of Psychology, University of Lisbon|Alameda da Universidade, 1649-013 Lisboa, Portugal; tmarques1@campus.ul.pt (T.M.M.); lcferreira@psicologia.ulisboa.pt (L.C.F.)

**Keywords:** parental alienation, family psychology, family court, custody issues, qualitative research

## Abstract

Parental alienation (PA) and its conceptualization or understanding of the process underlying this dynamic has long been controversial, but it has also been frequently brought to courtrooms. This study provides an account of how legal professionals conceptualize “parental alienation” and how they describe the characteristics of the phenomenon. Using a qualitative design, 21 family court judges (range 33–60 years; 11 men and 10 women), working with child custody cases, participated in an individual in-depth interview. A qualitative analysis based on Grounded Theory basic procedures revealed a complex picture of alienation dynamics with five interconnected results. First, PA contexts and landscapes, which included the judges’ perceptions on the PA nurturing contexts, its strategic behavior patterns and functions, portraits of PA and clues for its identification; second, considerations on PA severity; third, the influential factors, including those related to the emergence of PA; fourth, individual and relational impact of being exposed to PA; and fifth, perceived signs of change. The results also allowed for the complexification of the judges’ theories, revealing six properties of the PA concept: elasticity, intentionality and camouflage, power asymmetries, multifactorial nature, and destructiveness. Directions for future research are expanded from these results and pragmatic contributions of knowledge on judges’ critical thinking on PA issues and its manifestations in legal practice are discussed.

## 1. Introduction

The Parental Alienation (PA) field remains a stage for controversial critical reflections regarding its conceptual validity, admissibility, and use in family law [1]. However, an understanding of the factors influencing family court professionals’ decisions has not yet been achieved, hence delaying a robust scientific and legal basis to support child-centered and family-oriented law [2,3].

Parental alienation may be seen as an “international phenomenon” [4] (p. 428), found in courts and legal settings throughout different countries (e.g., in the USA, Canada, Brazil, France, Italy, Israel, Australia), and it has been fervently discussed in scientific and professional literature, encompassing different legal systems [4,5], policies and practices–e.g., see [6,7] for review. Portugal does not have specific legislation on PA; however, this concept is frequently brought to courtrooms and, as in other countries [8], legal professionals are quite familiar with and frequently make use of it. Hence, the close relationship between PA and Family Law has resulted in a frequent discussion of PA issues in cases of child custody disputes [9]. National institutions also provide documents and training events that seek to bring together the perspectives of law, psychology and psychiatry for a more comprehensive view of this phenomenon [10].

Despite the existence of some professional literature, academic literature is scarce on the underlying psychological issues and processes of family courts’ deliberations and, ultimately, the judges’ theories and meanings of PA, as well as their perceptions about specific needs in such cases. Thus, with this study we intend to fill this gap since further knowledge on this subject, through the eyes of legal professionals, may inform interconnected legal and therapeutic practices in cases of PA.

### 1.1. A Closer Look of the State of the Art 

Research focusing on professionals’ experiences and views, particularly those of family court professionals, remains scarce, although some studies have been conducted with both legal and mental health professionals. In this regard, some studies–e.g., [3,11]–have recruited family court professionals, including family attorneys, judges, therapists, psychologists, parenting coordinators, mediators, social workers, child custody evaluators and other clinicians, while other studies have included either specific groups of child custody evaluators—e.g., [12,13]—, divorce experts/mediators—e.g., [14]—, or therapists and psychologists—e.g., [15,16]. A recent systematic literature review [2] already reported some heterogeneity within the group of professionals working with child custody cases and PA cases, which leads to different professionals with different roles and functions being the focus of researchers’ interest. The authors uncovered a group of studies that underlined professionals’ perceptions of PA conceptual issues and their practical experiences working with PA. Through the results of the review, it is also noticeable that some studies have sought to understand the professionals’ evaluation processes, procedures and assessment tools, as well as practices and decisions in the field of child custody evaluation.

Given the historical association of PA with the notion of a diagnosable syndrome [17], initial PA studies sought to develop the conceptualization of parental alienation syndrome (PAS), its acceptance among professionals and validity as a syndrome [15], and to explore whether the symptoms of PAS could be empirically determined through professionals’ perspectives [14]. The studies targeting the PA or PAS concept and their underlying processes—e.g., [11,13,16]—mostly sought to explore the views, beliefs, opinions and experiences of professionals with regard to PA and PAS, in order to gain understanding of the changes within the professional field of child custody evaluation, namely those related to assessment tools and professionals’ practices or procedures. Professionals have reported knowledge about and familiarity with these two concepts [11,12,13,15] and also awareness regarding the controversies surrounding the PA-term and its association with Gardner’s work [11,16]. The conceptual enmeshment around the PA-term and the absence of guidance to work with these cases also appears to be a concern within the professional community [2]. Professionals who work as evaluators in PA cases reported using a range of tools and procedures for assessing PA dynamics and behaviors, including observations, clinical interviews, and a review of records and collateral sources [11,12,13], suggesting the need for a deeper understanding of the methods used by professionals in the context of PA assessments [13].

The qualitative research conducted by Viljoen and Rensburg [16] revealed that different dimensions within the legal system, namely the lawyers’ litigation attitude, may reinforce PA and pointed to the potential impact and emotional effects on professionals working with PA, such as self-doubt, disappointment, anxiety, and the risk of being confronted with ethical claims or malpractice accusations. These findings are in line with some ideas discussed by other authors—e.g., [2,18]—who highlighted the relevance of developing evidence-based assessment protocols and intervention programs in order to reduce the risk of claims and accusations against professionals.

Furthermore, due to the broader socio-historical context in which the PA concept emerged, it is not uncommon for it to be associated with allegation of sexual abuse [1], which has led some authors to develop empirical studies that use court databases or legal documents to analyze retrospectively decisions involving these two topics; for example, see [7,19] for discussion. More recently, other authors—e.g. [3]—recruited family court professionals and used vignettes with different case scenarios to research the effect of the alienating parent’s gender, child sexual abuse allegations and parental hostility claims on professionals’ evaluation of PA. The authors concluded that in scenarios presenting the mother as the alleged alienator plus allegations of parental hostility but no child sexual abuse, older or female court professionals were more likely to evaluate a situation as involving PA. 

In sum, although the scientific literature on this topic has presented a picture of PA where gender issues and child sexual abuse accusations—e.g., [19]—as well as domestic violence or IPV—e.g., [13]—appear to be relevant for decision-making processes, more in-depth knowledge regarding professionals’ theories and practical experiences with PA phenomenon is still sorely needed [2]. Studies addressing the factors that affect family court professionals’ evaluations are important for achieving a scientific basis for legal procedures and judgments [3], which reinforces the need to explore alienation dynamics, characteristics, roles, behaviors, and their associations with decisions and practical recommendations of court professionals.

### 1.2. The Present Study

As mentioned above, there is some literature on the experiences of PA-targeted parents (see [20] for a review) and on intervention programs, connecting both legal and therapeutic recommendations (see [21,22] for review). However, scientific literature regarding the beliefs, perceptions and lived experiences of professionals working with PA cases is scarce. As these actors play a fundamental role in the PA scenario, a proper exploration of their voices is crucial to further the advancement of the complex PA phenomenon field. The study of family court judges’ perceptions and experiences, along with their decisions and legal opinions is especially relevant to strengthen the existing knowledge within the academic literature and the professional community.

Accordingly, this study aims to explore the characteristics, processes and trajectories of PA from the perspectives of the decision makers, i.e., family court judges. Hence, the following research questions were defined: How do Portuguese family court judges conceptualize the PA construct? How do they professionally experience and theorize on the PA phenomenon? The answer to these research questions may be not only a relevant contribution to prevent misuses and misapplications of the PA concept but also a way of bringing together the languages of Psychology and Law, which may be an important step towards an intervention best suited to the needs of PA families.

## 2. Materials and Methods

### 2.1. Sampling Procedure 

The study included purposive sampling, i.e., the researchers searched for key participants with first-hand experience in the phenomenon under study. The main purpose was to access relevant data sources, thus increasing the breadth and depth of the data, rather than creating a sample with the intention of representing a population or making generalizations [23].

An in-depth search was conducted to locate Family Law Courts across the country and obtain the e-mail addresses of the judges working in each jurisdiction. The participants were then recruited through two complementary procedures: a formal request was made to the Supreme Judicial Council to authorize and disseminate the study to all Family Courts and judges; and invitations to participate in the interviews were sent directly to the judges through the e-mail addresses of all the Family Courts in the country. Participants and National institutions received no incentives for dissemination or participation.

The e-mail messages included the theme of the study, its general aims, the institutional contextualization as part of an ongoing PhD project at the Faculty of Psychology of Lisbon University and, finally, the invitation to participate in an individual interview focusing on parental alienation and legal professionals’ work in family courts. Fourteen Family Courts spread across seven Judicial Districts throughout mainland Portugal and the Azores agreed to participate. They forwarded the study’s information to the judges working directly with family law cases and requested the participation of at least one judge per judicial district. Twenty-one judges agreed to participate. After obtaining formal approval, the interviews were scheduled and carried out by the first author according to the participants’ availability. The judges were interviewed individually in their offices or in a meeting room at the court. The data was collected over a period of four months through in-depth semi-structured interviews to explore the participants’ perspectives, meanings of and professional experiences in parental alienation. All the participants were provided with detailed information on the study, namely the aims of the research and the details of participation—e.g., the average length of the interview; the right to refuse to answer any questions or withdraw at any time during the interview; data confidentiality; the request to complete a 13-item sociodemographic questionnaire at the end of the interview; email address of the research team for further clarification or contact. An informed consent document was signed by all the participants and their permission to use audio recording was obtained before the interview. All the interviews lasted around 90 min (range = 75–120 min) and were audio recorded and fully transcribed by the first author.

Ethical approval was obtained from the Ethical Committee of the Faculty of Psychology of the University of Lisbon before the data collection phase of the study and the ethical principles of the American Psychological Association [24,25] were followed.

It should be noted that, and in line with research quality criteria recommendations [23], before proceeding with the interviews, the first author conducted substantial preparation, through literature reading (e.g., scientific papers on family law and custody issues, technical and law-documents) to become familiar with judges’ legal jargon and meanings and with some of the key-terms, in order to explore the participants’ viewpoints in a fluid and experience-focused manner. Additionally, the first interviews were crucial as an opportunity to broaden the researcher’s knowledge of legal jargon.

### 2.2. Participants

The sample included twenty-one judges from Portuguese Family Law Courts. The participants averaged 47.57 years of age (range = 33–60 years; *SD* = 5.78) and the sample was comprised of a similar number of men (52.38%; *n* = 11) and women (47.62%; *n* = 10). Most of the participants were Portuguese (*n* = 20, 95.24%) and only one (4.76%) reported to have both Portuguese and French nationality. As for ethnical background, all participants identified as Caucasian/White (100%). Fourteen participants (66.67%) were married or in a civil partnership, four (19.05%) were divorced or separated, three (14.28%) were single, and 95.24% had children. All the participants had completed a university degree (80.95% held a bachelor degree, and 19.05% held a master degree). The majority (*n* = 17) had been practicing as family court judges for over 16 years (47.62% between 16–20 years; 33.33% over 20 years). The participants performed their professional activity in courts in several regions of the country (47.62% Lisbon and Tagus Valley; 19.05% Center; 14.29% North; 9.52% Alentejo; 9.52% Azores). Regarding religious beliefs, 71.43% were nonpracticing believers, while 9.52% were practicing believers and 9.52% were nonbelievers. Also, 4.76% were agnostic and 4.76% reported no religious belief. 

### 2.3. Measures

In line with the aims of the present study, the semi-structured interview guide included the following five main topics: *meanings associated with the PA concept* (e.g., “What, in your opinion, does parental alienation mean?”; “Bearing in mind recent cases, what signs led you to believe that you were facing a possible PA-situation?”); *PA and the legal system* (e.g., “After all the interviews with the judges have been completed, what would you expect to find in terms of consensus on the definition of alienation?”); *PA influencing factors* (e.g., “If we were looking for enhancers and obstacles of the PA process, what would we find?”); *severity of PA* (e.g., “If you were asked to create a PA severity scale, what levels would you set? And what would you use to describe and distinguish them?”; “If you had to advise a probationary judge, what would you say about the main contributions of the legal system to PA severity?”); *perceptions of change in PA situations* (“What signs are seen as indicators of parental and child change?”; “What key-moments have you identified as a turning points in the PA process?”).

At the end of the interview, sociodemographic information was collected through a 13-item questionnaire, which included questions on the participants’ age, nationality, ethnicity and religiousness, educational level, relationship status and children. They were also asked how long they had been practicing as family court judges and the region of the country in which they were located.

### 2.4. Data Analysis

The data analysis was carried out by the first author, despite constant discussion with the second and third authors, who are experts in qualitative methodologies. QSR Nvivo 12 software was used to support the analysis which followed the basic procedures and requirements established by the grounded theory methodology [23]. First, the researcher began the study with a theoretical background, having some prior knowledge of the literature in the field of PA before the data collection. The main researcher needed to be updated on the situation that would be studied and the full theoretical review performed is presented in [2]. Reflexivity was an ongoing process that guided the entire analysis, namely through memo writing and constant discussion among the authors. Writing the researchers’ thoughts, decisions, dilemmas and interpretations related to the research made it possible to assume this critical and reflective stance [23,26]. In line with the iterative process idea, data collection ended when the categories were saturated, that is, when new data no longer sparked theoretical insights, properties or patterns within and between categories.

After the full transcription of each interview, the analysis process of all the interviews was developed based on three main stages: initial, intermediate and advancing coding [23,27,28]. The analysis began with the initial (or open) coding, in which segments of the transcripts were labeled with a word or expression which emphasized the participants’ definitions of terms and implicit meanings. At this stage, the researcher engaged in a line-by-line coding and kept it close to the data. Specific codes were then organized according to main themes/categories, following the process of constant comparison to find similarities and differences between concepts and to search for patterns and relationships between categories—intermediate or axial coding. During this process, the main categories were identified and compared to others thus fostering the emergence of core categories. The resulting category tree not only identifies the core categories but also their sub-categories and interrelationships—advanced or selective coding.

## 3. Results

**Figure 1:**
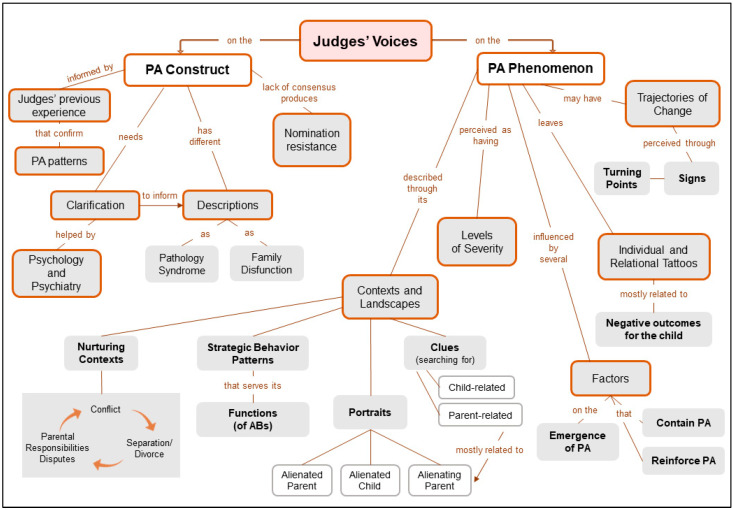
Judges’ voices on the PA construct and on the PA phenomenon.

In the Results section, categories and sub-categories resulting from qualitative analysis (Figure 1) are presented followed by brackets that illustrate the number of coded participants, whenever it is equal to or greater than one third of the participants (i.e., seven participants). Selected quotes aim to illustrate the judges’ perceptions, experiences and meanings of PA and are followed by: (1) one letter symbolizing the participant’s identification; (2) a letter representing gender (W—Woman or M—Man); (3) a range of numbers representing the age range of the participant. As an example, “(A-W41-50)” indicates a quote from judge A, who is a woman aged between 41 and 50.

### 3.1. Judges’ Voices on the Parental Alienation Construct

The results revealed unwavering beliefs regarding the PA phenomenon, sustained by the judges’ previous experience. The highly frequent use of some expressions throughout the judges’ narratives—e.g., “sometimes our intuition also guides us” (F-W41-50); “after many years of this (...) we almost guess what they are going to say” (D-M51-60); “this is often a pattern too!” (N-M41-50)—points to the idea that *the judges’ previous experience* is used to confirm *PA patterns*. According to the judges, there is a consensus that PA really exists. However, the controversy regarding conceptual and nomination issues was also mentioned—“it’s a very real, very present reality, yet it’s a term we avoid (…) the problem begins with ‘How can we name it?’” (N-M41-50). In this regard, the idea emerged of the PA term as a “bag” with diffuse and poorly defined boundaries—“it’s a term that people use without due rigor, where everything fits! So it’s a rather large bag (laughs)” (O-W41-50)—which seems to be connected to the idea that the PA concept needs further scientific *clarification* (12) through the *help of psychology and psychiatry* (8), which illustrates the contribution of sciences other than law: “Then in technical terms, knowing whether this is parental alienation, I must confess that the idea I have is that this is something more related to psychology than to law” (B-M41-50).

The lack of consensus regarding the conceptualization of PA was clearly evidenced in the participants’ narratives, as some of them referred to it as a *pathology/syndrome*—“(…) there will be some psychiatric disorder (…)” (J-W31-40)—while others highlighted it as a *family dysfunction*—“It will be the result of the configuration of certain family relationships, in certain situations” (U-M41-50).

In some participants’ discourses, *nomination resistance* (7) was also noticeable, with other-related concepts being used by the judges to replace the PA term. This resistance–referred to by a participant as “name syndrome” (A-W41-50)—may be rooted in the historical origins of the concept and in the negative connotation associated with it, generating theoretical discussions that overlap with the essential discussion on the substance.

### 3.2. Judges’ Voices on the Parental Alienation Phenomenon

#### 3.2.1. Looking at Parental Alienation—Contexts and Landscapes 

**The Nurturing Contexts.** The analysis revealed three main interconnected ideas that describe the context in which PA processes take place: *conflict* (19), *couple separation or divorce* (18), and *parental responsibilities disputes* (18)—“If the pattern of relationship between those parents is one of great conflict (…) complaining to the court because a parent was five minutes late bringing [the child] back or because the children were left without their sneakers (...)” (O-W41-50). Alienation feeds on conflict, which appears to manifest or intensify itself during or following the couple’s separation/divorce. During this period, family dynamics change and parental responsibilities have to be regulated by the court. Hence, parenting disagreements are frequently exacerbated.

The participants also emphasized the *socioeconomic context* (8), referring to the relationship between higher economic, educational and social status and higher litigation (high-conflict cases), which typically supports more severe levels of PA.

**Strategic Behavior Patterns.** The results shed light upon alienating behaviors (ABs) enacted by the alienating adult over time. According to the participants, ABs configure an ongoing strategic pattern through which the alienating adult intends to damage the relationship between the alienated figure and the child. All the judges (21) mentioned an AB network, as described below.

*Making False Accusations against the Other Parent*. This was mentioned by almost all the judges (20) as one of the main strategies used to justify breaking the other parent-child contact. The data analysis revealed that the most common accusation is *sexual abuse* (19). Some judges (8) highlight that the frequent use of sexual abuse accusations was, for a long time, largely associated with the perception of immediate gains, such as the suspension of the child’s contact with the accused parent: “It [sexual abuse] is the kind of story that immediately makes all the ‘bells’ ring, and, therefore, everyone acts to break off contact with that parent (…) when in doubt, let’s break off the relationship!” (O-W41-50). The participants also mentioned *accusations of negligence, poor parenting skills, domestic violence* and *maltreatment.*

*Blocking Communication*. According to the judges (17), blocking communication corresponds to a range of actions—both direct and indirect—that aim to limit the time and participation of the other parent in the child’s life. The most mentioned direct actions were *non-compliance with court orders on non-resident parent-child contacts* (9)—“When we decided that the non-resident parent will pick up the child from school to prevent this type of thing, the child doesn’t go to school on that day (...)” (M-M41-50)–and *moving away with the children* (9)—“I have a recent situation in which both [the two parents] lived in V., then, one of them went to another more distant city, making it difficult to keep in contact” (P-W46). Some other participants also mentioned *limiting participation in the child’s routines/rituals*, and *hiding relevant information about the child*. Less mentioned were *claims of illness on visitation days, creating obstacles to the judge’s proposals,* and *asking for progressive visitation arrangements or free parenting plans.*

*Devaluing and Displaying Contempt towards the Parenting Role of the Other Parent*. This pattern, mentioned by around half of the participants (15), included references to: encouraging the child to call another adult “mother” or “father” or denying the existence of the other parent; undermining the parental image and minimizing his/her authority by taking unilateral decisions or devaluing the other parent’s educational preferences; and depreciating the other parent in front of the child.

*Gaslighting*. Some participants (11) have emphasized strategic ABs corresponding to the presentation of false or distorted information with the intent of making the child doubt his/her own memory or perception, thus influencing his/her view of the other parent—e.g., telling the child falsehoods/lies or incomplete things about the other parent, such as telling the child untrue or made up stories or that the other parent doesn’t love or care about him/her.

*Terrifying the Child*. A small number of participants also identified some behaviors that intend to persuade the child that the other parent is a very dangerous person and, even threatening the child with the withdrawal of love and affection if he/she does not comply with this vision.

**The Functions of Alienating Behaviors.** Thus, these patterns and strategies point to three main functions of alienating behaviors (ABs), namely: *undermining and preventing the parenting role of the alienated figure and his/her relationship with the child* (20)—“(…) [It] is trying to create a negative image of the other parent, so that the affective connection between them is lost” (P-W41-50); *controlling the alienated parent’s access to the child* (20)—“The father says that he has tried in every way to be with the children (…) and that the mother has always obstructed these visits” (E-W41-50); and *having control over the child*, promoting his/her loyalty and dependency (15)*—*“they manipulate and make children take positions in relation to the other” (I-W41-50). These functions can be seen as being in a feedback circuit—a circular and non-linear system—in such a way that the aforementioned ABs can serve more than one function and these functions feed on each other to contribute to PA.

**Portraits of Parental Alienation**.

*The Alienating Parent.* With regard to the portrait of alienating parents, the judges stated that *the custodial/resident parent is most often the alienating parent* (15) and *that more mothers are alienating parents than fathers* (15), which is associated with the historical-socio-cultural role of mothers as primary caregivers. Hence, in separation/divorce situations, mothers are still more likely to be the custodial parent, thereby having control of the logistics required to alienate the child—“(...) in the overwhelming majority of cases the alienating parents are mothers (…) this is also related to the role of women in our society (…) she is the caregiver!” (A-W41-50). Hence, this idea appeared to be closely related to two organizing beliefs identified in the judges’ narrative: mothers are better (and the only needed) providers for the child than fathers and fathers do not have enough parenting skills to take care of children—“In a couple relationship, sometimes fathers have more of a back-office attitude (...) when they [mothers] get here, it’s ‘Oh! But he doesn’t know how to do any of that!’” (O-W41-50).

It is also worth noting that, although the reference to mothers as preferred alienators is not always explicit, the examples provided by the participants generally involved an “alienating” mother and an “alienated”/“rejected” father, such as “In general terms, mothers think that (…)” (J-W31-40), which demonstrates the gendered nature of the parental alienation discourse.

Alienating parents were also described as “tentacled [octopus]” (O-W41-50), an idea that can connect several characteristics attributed to these parents: being possessive and controlling (13); self-centered (10); and inauthentic, dissimulated and manipulative (8).

First, alienating parents were referred to as *being possessive and controlling*, acting as the owner of the child and as the owner of the truth about what is best for the child:

*(…) a sentence that is often mentioned is “I let my son go to visit his father!» as if “letting” was because [that parent] has the power to not let! It is even necessary to deconstruct that idea of the resident parent as if she/he were the owner of the child!*

(S-W41-50)

As with an octopus that is clingy and has many tentacles, it is as if the alienating parent has a need to control and influence everything that concerns the child and his/her contact with the other parent.

Moreover, also mentioned by some participants was the idea of alienating parents as *self-centered*—“Very self-centered on his own needs, the need to have his daughter in his care, and not very focused on his daughter’s interests...” (S-W41-50)—and using the privileged position of resident and closer parent to alienate the children and, consequently, maintaining the position of power and control and satisfying his/her own aims. Thus, just like a manipulating octopus which is, by definition, self-centered as it pulls everything to its center, alienating parents are perceived as being more focused on their interests, desires and motivations, and less focused on the children’s needs.

Some judges also referred to alienating parents as being *inauthentic,* *dissimulated* (in the sense of false cooperation), and *manipulative*. This description unveiled the idea of a “camouflage game” that happens when the octopus uses camouflage by changing its appearance and its way of moving to manipulate others’ perceptions:

*(…) when the parents arrive here, the mother’s attitude towards the court is one of total collaboration. (...) because the mother says “Yes, yes! But he [the child] can be with his father, he can call whenever he wants.» And when she leaves the courtroom it is just the opposite!*

(T-W41-50)

*The Alienated Parent*. Regarding the alienated parent, the results pointed mainly to *inertia and the passivity pattern* (8) that reinforces PA. First, this portrait included the tendency to assume a more secondary role in the child’s care and education, with the most prevalent idea of fathers as the alienated parent. Second, the judges highlighted the alienated parents’ tendency to react with inertia to the difficulties of contact with the child during or after the couple’s separation and maintaining a passive attitude—“when you take a closer look, they didn’t make that much effort!” (E-W41-50). Some of the judges also described these parents as not being persistent in the face of the child’s resistance or refusal and highlighted withdrawal and counter-rejection as common patterns of the alienated parents.

*The Alienated Child*. According to the judges (12), alienated children display “serious psychological distress” (A-W41-50), particularly sadness, difficulty in verbalizing their own feelings and dependence on the alienating parent. Some of the judges also mentioned children’s binary thinking tendency “bad parent vs. good parent” as a pattern—“Whenever there is an alienating parent, the child loves the alienator and does not know that she/he is alienating! (...). This parent is fully supported!” (H-W41-50). Some of the judges referred to the relevance of the children’s developmental stage, suggesting that early ages are associated with higher vulnerability as the affective bonds with the alienated parent are not sufficiently consolidated and the cognitive mechanisms are not as developed as in older years.

**Parental Alienation (In)Visibility—Searching for Clues.** All the judges identified child-related (18) and parent-related (20) clues that place them on alert for the possibility of PA. As regards *child-related* clues, the *child’s contact resistance or refusal*, particularly if it appears associated with sudden and/or unjustified rejection, emerged as the most highlighted—“If the child is unable to explain why he does not want to be with his father or mother and does not give me any plausible reason, I am immediately on alert!” (A-W41-50). The *low level of authenticity of the child’s discourse* was also identified as being related to a “second-hand discourse” (D-M51-60) with arguments glued to those presented by the alienating parent, a “very well prepared, very well trained speech” (F-W41-50) with little spontaneity; and an adult-like speech “with vocabulary that children do not usually have” (F-W41-50), that appears to mimic what they have heard an adult saying. According to the participants, the child’s reports of events and memories can also be a relevant sign when pointing to *questionable memories* as they could not have been experienced or remembered by the child at the age that she/he would have been at that time—“He remembered that at 2 years old his father had given him half a burger, but his freezer had been full!” (T-W41-50).

The results also shed light upon *parent-related* clues, which mostly included references to the *alienating parent*. The participants reported being attentive to *common arguments* that are brought by parents in cases of PA, such as “[the parent says] ‘she/he doesn’t want to go [to the other parent’s house]! As far as I’m concerned, she/he can go! But she/he doesn’t want to’” (J-W31-40); and to discourses that suggest that *these parents perceive themselves as the child’s owners*—“[the parent says] ‘I never forbade him/her to see the child!’ and I say ‘What if you did forbid him/her? You have no right to forbid!’” (D-M51-60). Some judges also mentioned other parental behaviors such as *non-compliance with court ordered parenting times, presenting obstacles to the child’s contact with the other parent* and *little openness/resistance to the judge’s proposals*. It was also mentioned *making allegations or raising suspicions*, especially those related to sexual abuse or the other’s skills to care for the child. A few judges also pointed out two other parent-related clues, such as *non-resident parent complaints to the court* (e.g., contact with the child is proving difficult; the resident parent does not allow this contact) and the *high level of parental conflict* (with possible refusal to talk to each other).

#### 3.2.2. Severity of Parental Alienation

The participants’ narratives revealed three *levels of severity*. As regards the *high level* of severity, the judges emphasized the use of false sexual abuse accusations as a way to hinder the access of the other parent to the child—“I think the most serious accusation that can be made of a parent is that he[/she] sexually abused his[her] child” (H-W41-50). The participants also mentioned other alienating behaviors, such as moving away or relocating to another country, since “there is a total absence of contact” (J-W31-40). In addition, this level of severity also included references to the child’s explicit and total refusal to be with a parent and a “closed attitude” to the contact-oriented intervention.

*Middle levels* of severity were mainly associated with: specific alienating behaviors such as maltreatment or neglect accusations; partial non-compliance with court-ordered parenting responsibilities; and an initial definition of the child’s preference for non-contact with one parent, which means “(…) that contact can be avoided, but there is no aversion, as such” (U-M41-50).

The judges related the *low level* of severity mostly to behaviors that belittle or devalue the other parent—“[This is] less severe because (...) it only induces the idea that one is more capable than the other, ‘Your father was never capable of this...’ or ‘It was always me who changed your diapers...’” (P-W41-50). The existence of contact, even if intermittent, was also mentioned at this level of severity, and the child’s openness to parenting schedules proposed or ordered by the judge. 

#### 3.2.3. Influential Factors on Parental Alienation

**Influential Factors on the Emergence of PA.** The results revealed three strongly interrelated themes regarding the influential factors for the emergence of PA: *Relationship rupture and resentment* (18), *Clean slate* (11), and *I am the one* (11). Additionally, two other themes emerged: *Alienator’s personality and childhood history* (8), and *A dangerous father.*

*Relationship Rupture and Resentment*. This theme refers to the most frequently mentioned factors, which were mostly related to the end of the relationship as a couple and to the associated negative subjective experience (e.g., feelings of rejection; resentment; anger; difficulties with emotional grief)—“(…) it is connected to the end of the relationship between the parents (...) the one who is dismissed, is the one who is hurt, carrying great grief and unable to deal with the situation” (F-W41-50). Some of the judges also pointed to the financial issues that come with separation/divorce as contributing to the emergence of PA, namely non-payment of child support was identified both as a trigger and a consequence of PA, creating a dysfunctional cycle where payment and contact are used as a “bargaining chip”.

*Clean Slate*. This theme is closely related to the couple’s separation and includes references to the desire to start over, or start a new family with a new partner and, in some cases, excluding the other parent—“(…) because they come to the conclusion that [that person] was a mistake in their life” (L-M51-60). However, a new beginning was also perceived by the participants as enhancing the trigger factor when there is a new partner— “(...) perhaps some degree of sorrow due to the other having re-started his/her life. And then, [he/she] starts to make the contact difficult” (J-W31-40).

*I am The One*. The judges highlighted the influence of gender role differences stressing the mothers’ positive self-evaluation of their parental role, associated with their critical view of fathers having fewer parenting skills and being less capable of ensuring their children’s needs—“(...) they [mothers] feel they are the only ones who can look after their children properly (...)” (A-W41-50).

The judges associated this deep-rooted role with the way mothers perceive fathers who engage in parenting tasks and functions as a threat to their caregiver role:

*(…) there are a lot of mothers who cannot cope with situations where the father wants to take a more active role (...). [They] feel to some extent that “he is stealing my place and he is having the same role in the child’s life that I have, but I am the mother!»*

(F-W41-50)

*Alienator’s Personality and Childhood History*. Some of the judges highlighted particularly the need to understand each parent’s individual attachment style based on their childhood and family of origin experiences— “(…) this alienating mother wants her child for herself alone, not to take her father away, but because she has an obsession with her child (...). It is more a disorder of the mother herself” (T-W41-50).

A Dangerous Father. The alienator’s perception of the other parent as a dangerous person—“(…) we realize that this person really sees the other parent as horrible and monstrous and that she/he will only harm the child!” (O-W41-50)—has also emerged as a possible PA influential factor.

**Reinforcing and Containing Factors.** According to the judges, there are several sources that contribute to reinforcing or to containing PA: time, the legal system, the alienated parent’s behavior, the extended family, the social network and the intervention. 

*Time* (13) refers to the sluggishness of legal proceedings, length of exposure to alienating behaviors and duration of the parent-child absence of contact, and it is seen as a strong ally of PA, opening up a space for the PA process to unfold:

*I saw that there was already PA and the fact that the case took a long time to reach the trial stage, because social services reports are needed, prolonged the parent-child separation. Therefore, the mother continued with her PA battle! PA is not caused by the sluggishness of court proceedings or the entry of the cases in the court system, but of course, the legal proceedings themselves exacerbate the desired effect of PA, which was already there anyway.*

(T-W41-50)

In contrast, a few participants pointed to the containing role that Time can also play, as it allows for the establishment of distance from situations that are at the root of PA. 

The PA reinforcing role of *legal system-related factors* (14) was mostly related to the judges’ procedures and decisions throughout the legal process, namely forcing a consensus to reach a written agreement on parenting time schedules—“(…) imposed decisions, that is, when they are not consensual and people have to continue to live with one another, often end up serving to further exacerbate the conflict” (R-M31-40); or suspending parent-child contact in cases of accusations—“(…) a bell rang and it was over... Visits are suspended! And this was exactly the [other parent’s] intended goal” (H-W41-50); or hearing the children in court on several occasions, since “the systematic verbalization of the facts causes the story to become anchored and take on the contours of reality, even if it is false” (A-W41-50); or leave the case without a temporary decision, while awaiting specialized technical evaluation. Legal system-related factors were also related to lawyers’ adversarial stance, which perpetuates the win-or-lose vision instead of encouraging cooperation between parents.

Notwithstanding, the participants also emphasize the containing role of some legal system-related factors (15), namely, the judges’ procedures (e.g., responding promptly, as a way of acting in the early stages of the process to avoid crystallization; prioritizing solution pursuit action; following-up on their decisions, acting in close proximity throughout the process; investing in deconstructing the parents’ distorted views and promoting parental reflexivity) and decisions (e.g., settling shared residence as a solution that balances the power between the parents and reduces inter-parental conflict; establishing temporary decisions to remain in effect until the final ruling, aiming to reduce the decision-voids and the margin for PA to occur).

The analysis also revealed that *alienated parents’ negative behaviors and attitudes* (9)—e.g., giving up or divesting in their attempts to restore the relationship with the child, inertia or passivity, counter-rejection of the child, self-centered or immature attitudes, and rigid parenting style—are identified by the judges as playing an important role in the maintenance of the PA dynamic, confirming the alienating parent’s beliefs, and even the child’s negative views.

The *alienated parents’ positive and invested attitude* emerged in the discourse of a few participants as a contribution to containing the PA process, particularly being empathetic, since this attitude can reduce potential hostility, and being persistent.

The extended family influence as a PA reinforcer was also mentioned by the judges, particularly the *pro-conflict family position* (7) which included references to the extended family’s “campaign” which supports and encourages one parent’s ideas while denigrating the other parent. However, the potential buffering role of the extended family was also highlighted by some of the judges—“A family support network that shows the alienating parent that this is not the way” (T-W41-50). The analysis also revealed the peacekeeping role of *social network* (7), i.e., each parents’ supportive and close relationships, acting as “blue helmets” (P-W41-50) and contributing to PA containment. With regards to *intervention system-related factors* (9), the judges mentioned the relevance of technical/professional support beyond the context of legal action, such as psychotherapy, psychological support, family mediation, parental counseling and education, or family therapy—“I think the only thing that gets to the heart and bottom of this issue is family therapy”(E-W41-50)—and highlighted the need for a prompt response/action by these services since “if it is not stopped at an early stage, then it can infect and create irreversible damage” (O-W41-50).

A few of the judges also mentioned other reinforcing factors, such as the *influence of the media,* namely the reporting of problematic cases, *parents’ resources for litigation* and *the perception of impunity*, the latter two bolstering the alienating parent’s power. 

#### 3.2.4. Parental Alienation Impact: Individual and Relational Tattoos 

The analysis revealed that PA is mostly perceived as being considerably harmful and having “a tendency to crystallize” (D-M51-60) while it also “(…) leaves many negative marks” (U-M41-50). More than half of the participants mentioned *negative outcomes for the child* (16) and pointed to *other possible outcomes* (17), which included references to the alienated parent or to the family relationships.

The judges pointed to the short-term and long-term adverse effects resulting from the child’s exposure to PA. As regards negative short-term consequences, the participants identified psychological and emotional suffering, distrust and fear of relating to others, physical-medical symptoms and behavioral changes—“These youngsters, in general, grow up with great anger within them. There are a number of feelings that they don’t understand, they can’t deal with” (U-M41-50). Distrust and fear emerged in association with *the view of the world as a dangerous place*: “[The child] had loads of phobias with the world! It wasn’t just her father... It was related to big dogs, she had phobias regarding her peers, streets, lots of different things” (C-M51-60). The construction of children’s sense of identity can also be negatively affected due to the impact of the PA situation on their family representation.

The participants also described long-term consequences, such as for mental health, as well as the possibility of repeating the relational patterns experienced during childhood: “These people, one day in the future, are also likely to become alienators...” (I-W41-50).

As other possible outcomes, the participants identified the impact that alienation dynamics have on family relationships as a whole and, particularly, on the parent-child affective bond—“It [PA] affects the child (...), affects the parent who is alienated, but it also affects the alienating parent” (L-M51-60). They also emphasized the emotional fatigue and exhaustion that PA causes the alienated parent, as well as resignation as a means of protecting the children from suffering. In PA situations, the lack of parental alliance may also lead to role reversal in the family structure, more specifically with the *Adultification of the child* (12). This dysfunctional family pattern implies considering the child as being in an adult position and responsible for making decisions. Some of the participants mentioned the pressure that is placed on the child to make decisions on contact with the other parent that please the alienating parent—“they say ‘you know, talk to your father if you want to. If you don’t want to, don’t talk to him!’ (…) We are talking about 7 to 8-year-old children” (B-M41-50). Other participants also referred to sharing adult information with a child, such as financial or couple relationship information, as a concern in PA cases.

#### 3.2.5. Signs of Change: Looking for Turning Points 

Although, as mentioned, PA is mostly perceived as having “a tendency to crystallize” (D-M51-60), some of the judges (11) identified specific signs that help them perceive a trajectory of positive change in the PA process: more positive and spontaneous child’s attitude towards the alienated parent; greater receptiveness to have contact with that parent; improvement in school performance; and increased tranquility—“[the child] becomes more relaxed, with better grades at school... Talks more openly... So these are all signs that something has been restructured in that relationship” (T-W41-50). According to the judges, signs of change in the parental couple—e.g., improvement in parental communication, more trust and flexibility as opposed to control, and more active and involved participation of the alienated parent in the family’s life—can also indicate the possible dissolution of the PA trajectories—“when the resident parent begins to listen to the opinion of the non-resident parent, for example, starts to respect his/her role, ceases to create obstacles (...), begins to trust... (...) then I start to think ‘this is already reversing’” (A-W41-50).

The participants mentioned other signs, such as the “parents’ openness” (Q-M41-50) to the judge’s proposals (e.g., for parenting arrangements, contact supervision or intervention); more compliance or fewer lawsuits brought to court; and feedback provided by the services intervening with the family (e.g., the contact between the child and the parents is described as being more positive; proposals to change the parent–child visitation/parenting plan, increasing contact time between them due to an improvement of the relationship).

*Turning Points*, acting as stepping stones for positive change in PA cases, were also identified (9): parental change leading to a change in the child’s resistance; diagnosis of serious illness in the child; parents tiring of being in constant conflict; the main alienating figure being removed from the child; change of residence during adolescence; an unexpected encounter between the child and the alienated parent in court; the alienating parent observes the child’s positive relationship with the other parent; positive relationship with new partners; and “giving voice” to adolescents in a resistant position.

The participants’ discourse on positive change and the turning points in the PA process sometimes emerged associated with the idea that “[these changes] always end up being very volatile situations” (J-W31-40), which points to the difficulty of maintaining a positive evolution and achieving stable changes in the parent–child relationship—“If there is something that at any point does not go well, we quickly go back to square one” (J-W31-40).

## 4. Discussion

**Figure 2:**
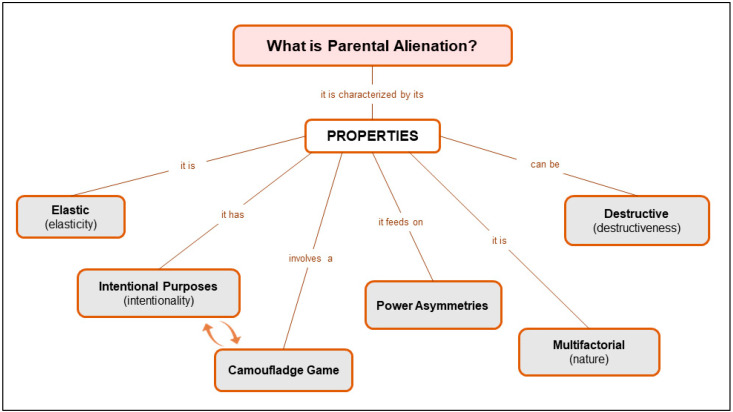
Judges’ theories on the PA construct.

The present study sought to ascertain, through a qualitative approach and guided by the methodological principles of Grounded Theory, how family court judges conceptualize the PA construct and how they professionally experience and critically reflect on PA characteristics, processes and trajectories. The judges’ perceptions of the mitigation and recovery process also emerged through data analysis (Figure 2).

### 4.1. Grounding Judges’ Theories on the Parental Alienation Construct 

Despite the controversy regarding the nomenclature and the debates around its validity or formal recognition as a diagnosable syndrome–e.g., [29,30], there is no doubt that PA has been the target of scientific and professional community interest. In line with what other authors have referred to in their publications–e.g., [5,8], the participants of this study also reinforce the idea that, regardless of its name, nature and characteristics, PA is a real problem which they observe every day.

A holistic and reflective interpretation of the results leads to an in-depth comprehension of judges’ theories on the PA construct. Thus, the results suggest six properties of the PA construct, i.e., characteristics or special attributes that provide meaning to it, namely: elasticity, intentionality and camouflage, power asymmetries, multifactorial nature, and destructiveness.

**Elasticity.** The idea of PA as being elastic is connected to the diversity of concept descriptions and to the fact that different terms—such as ‘parental alienation syndrome’, ‘parental alienation’, ‘alienating behaviors’ or ‘child instrumentalization’—are used interchangeably by the participants, which is also a concern raised by previous studies, for example, [6,31]. Whether due to its conceptual enmeshment, or due to the judges’ lack of knowledge of psychological jargon, the PA term appears to have sufficiently elastic boundaries to include a variety of situations that the judges perceive as “parental alienation.”

On the other hand, the manifestation of PA, in the sense of its elastic capacity to expand socially and scientifically, appears to depend on the “space” offered by the socio-cultural context. Over the last few decades, the scientific and legal literature has sought to describe this phenomenon internationally, and some authors believe that PA transcends politics, culture and religion—e.g., [4,5]. However, depending on the social, cultural, economic and political reality of each country or state, different “visibilities” may be given to the PA concept and to the PA phenomenon. The results of this study point to its greater expression or visibility in urban areas, where there are more economic and social resources to prolong litigation and where the evolution in the role of women in the family-work interface and the transformation of men’s role as fathers in today’s society are more accentuated. These contextual differences lead us to consider the possibility of the phenomenon manifesting itself differently in societies and contexts in which there is little to no social, cultural or legal space for parental alienation—whether due to issues of poverty or social vulnerability; in patriarchal societies that place women on a lower rung within the family hierarchy; or in countries wherein the court system does not have a legal lexicon or an equivalent term to that of the English term ‘parental alienation (syndrome)’ (e.g., the Islamic legal system in Israel does not have a parallel concept) [32]. If there is no manifestation of the phenomenon in certain locations, then it could be more a matter of being faced with a “non-defined-problem” than the actual absence of PA dynamics.

Finally, and at a more micro level, this property—elasticity—emerged in liaison with the idea of the extended family as a “secondary alienator” that encourages or supports the alienating parent’s ideas, behaviors and positions, or, even, may assume the alienating role often assigned to parents. Additionally, PA can be extended to the alienated parents’ extended family, which is “caught” in the middle of this elastic alienation dynamic.

**Intentionality and Camouflage.** A closer look at the specific categories described in the results section—i.e., strategic behavior patterns; alienating behavior functions; PA portraits-the alienating parent; and PA (in)visibility, looking for clues—sheds light upon two relevant and inter-connected properties: intentionality and camouflage.

Intentionality as an emergent property illustrates the conscious, strategic and/or purposeful malicious nature of the alienation process, while camouflage points to a dimension of PA as a “dirty game”, which is characterized by non-transparency and secrecy regarding these intentions. As highlighted in the literature, the results suggest that alienating behaviors (ABs) contribute to a child’s alienated stance [33] and are at the service of certain functions, i.e., these behaviors are commonly used with the intent to harm the other parent or to interfere with the child’s relationship with the other parent, for example, [34].

At the same time, these alienating parents—who are, not infrequently, the child’s preferred parent—could be seen as making “seductive invitations” to the child to take sides. They can be perceived as “seductive” since they “intentionally invite” the child to a privileged, stable and protective relationship, which apparently does not exist on the other side. The alienating parent can, thus, be “two-faced,” being a devoted and consistent parent towards the child while, simultaneously, interfering in the child’s relationship with the other parent [34]. This audacity and non-transparency are also connected with the idea, expressed by some of the judges, of alienating parents being tentacled [octopus]. The idea of exercising power and control over the child and over the other parent, while camouflaging their way of acting, their intentions and their non-collaboration with the judges’ proposals, appears, according to the judges, to be at the heart of PA dynamics.

The results described in Parental Alienation (In)Visibility—Searching for Clues also seem to contribute to highlighting these two properties—intentionality and camouflage— as most of the clues point to the inauthenticity of the discourse and the obstacles (not always clear and direct) that are created in the face of the judges’ proposals and, above all, to the child’s resistance to interactions with the rejected parent. Once again, although these signs may be clues to a PA dynamic, there is always the possibility that hidden intentions and camouflaged actions make the formal recognition of PA difficult.

**Power Asymmetries.** This property emerged from the results conveying the idea that PA “feeds on” asymmetries in the relationship of each parent with the child (or in the access to child-related information), and from the participants’ belief that some factors may function as boosters of these power asymmetries—e.g., decisions that favor one parent (e.g., primary or sole custody); suspension of contact in the face of the alienating parent’s accusations; involvement of other professionals, such as lawyers, who, by having a “litigation culture” instead of a “conciliation culture” can strengthen the alienating process. In line with what has been mentioned by other authors, such as [16,35], our participants identified the contribution of the legal system in creating and exacerbating power asymmetries, particularly through court orders that provide unequal parenting time to one parent over the other. Harman and colleagues [35] also mentioned that some imbalances in power between parents may be related to specific behaviors of the alienating parent (e.g., coercive controlling strategy patterns; loyalty inducing behaviors targeting the child).

The judges also referred to the alienated parent’s sense of powerlessness and the alienating parent’s perception of impunity. Court times and formal proceedings slow down the custody process, which provides the time needed by the alienating parent to alienate the child—e.g., [16,33]. Additionally, the alienating parent’s perception of impunity, in cases of non-compliance with the court-ordered parenting time schedules, reinforces these asymmetries, since the alienating parent acts as if she/he is above the law and immune to punishment. In this context, the information and relationship that the alienated parent establishes with the child is highly dependent on the alienating parent, but this does not occur in the opposite direction. In fact, in order to obtain control, the alienating parent may try to use coercion or controlling behaviors [34,35]—for example, through manipulation of the system or limiting the alienated parent’s access to information about the child — thus decreasing the power of the other parent, creating more accentuated asymmetries in the relationship with the child and creating the child’s negative perception of the alienated parent.

**Multifactorial Nature.** An in-depth examination of the results reveals the notion of PA as multifactorial, i.e., caused by an interaction of several factors and processes, which is in line with what has been theorized in the literature on PA assessment and intervention—for example, [1,33,36]. Firstly, the analysis of PA nurturing contexts highlights, from the outset, a facilitating interaction—a “perfect triangle” between high-conflict, couple separation and child custody disputes—which, once established, facilitates the emergence of PA processes—for example, [16,37,38]. Furthermore, according to the participants’ view, the alienation process can be better understood through the multiple and interrelated factors that contribute to containing or perpetuating these family dynamics. This is in line with the literature which indicates that not only should alienating behaviors (ABs) be considered to determine PA, but also other relevant factors, including the children and alienated parents’ psychological and behavioral responses. The current findings are also coherent with previous descriptions of targeted parents as being rigid, passive and trying to cope with the child’s rejection by withdrawing or with counter-rejection—for example, [33,36].

**Destructiveness.** The results of this study also shed light on the destructive nature of PA, which is in line with previous research on this topic [16,39]. This property emerged as the different themes identified in the results appeared interconnected to explain how this phenomenon can be highly destructive and to define severity levels based on individual and relational impacts. Consistent with previous literature describing ABs as behaviors that control, coerce, and generate fear in the child [34], our results revealed the idea that the world—beyond the alienating parent-child “bubble”—is anxiously experienced by children as dangerous and threatening to their safety. As other authors such as [34] have noted, some ABs may have the potential to distort the boundaries in the parent-child relationship, while complete loyalty is demanded from the child, who is placed in the position of ally. Over time, destructive processes may occur, with adultification, parentification and infantilization as possible paths [40] that promote and enmesh the parent-child dyad, where the needs of the child are not met.

The PA phenomenon was also described by the participants as tending towards “crystallization”—in the sense of its continuous increased severity—with little room for recovery trajectories. In association with the destructiveness property and the judges’ ideas on the mitigation and recovery process, the time dimension emerged. Not being defined as a property per se, its relevance is unquestionable when trying to understand the complexities of PA processes. The child’s prolonged exposure to ABs, the duration of the child’s separation from the alienated parent, and the sluggishness of legal procedures all share the notion of time; more precisely, the idea that the longer it takes, the more destructive it will be for the child and for the entire family system.

At the same time, the positive evolutions and stability of the positive changes achieved are also described using the time dimension, as they are perceived by the participants as sporadic or short-lived, especially in more serious situations. However, and as previously identified by other authors such as [41], the judges recognized that the destructive effect of time could be decreased or mitigated through early intervention and prompt response when at-risk cases are identified by the legal system.

### 4.2. PA Phenomenon through the Judges Windows

The PA process, as conceptualized by the judges, seems to need a systemic and interconnected view of all the factors and dynamics that contribute to its development, maintenance and mitigation. As such, the judges’ “windows” uncovered a “nodal landscape” composed of multiple factors and complex processes that transcends looking at each individual—mother, father, and child—separately, contrary to the linear and simplistic idea that only the actions and attitudes of the alienating parent affect the child’s contact refusal and perception of the alienated parent. As stated by Garber [42], it is more a matter of “dynamics, not diagnoses” (p.368), and PA is more about the relationships and system that these individuals make together than about individuals and the description of behaviors. 

In the current study, other “peripheral landscapes” began to take shape as the judges described the parental characteristics and contexts that facilitate alienation dynamics. The judges highlighted the tendency for mothers to be in the alienating parent’s role, which may be associated with their historical–socio-cultural role as primary caregivers, shedding light on the mother’s perception of the other parent as a dangerous person, as having fewer parenting skills and/or as being less capable of ensuring their child’s needs. While this may be a strong and unsubstantiated belief of mothers, it is also possible that, in some cases of PA, the alienated parent contributed to the alienation in one or more significant ways. This possibility leads us to the landscape of “hybrid cases” [36], which identifies a combination of both parents contributing to the alienation of their children. Hybrid cases allow us to understand the child’s rejection through a combination of alienation and estrangement elements, with the child’s exposure to alienating behavior from a parent, but also with the child’s direct experience of abuse, neglect or real caregiving deficits of the other parent. On the one hand, the advantage of looking at this concept of hybrid cases is that in addition to black and white cases, it is also possible to frame gray areas and, on the other, it has implications, such as the need to balance the supposed alienation of parents who seek to protect their children.

### 4.3. Limitations, Strengths, and Pragmatic Contributions

Although the present study provides important contributions to the PA scientific field, some limitations must be acknowledged.

Family court judges were intentionally recruited for having first-hand experiences in PA and for being the best informants for this study; however, it was clear during the interviews that judges from other courts—e.g., from courts of general jurisdiction and not just from family jurisdiction—as well as public prosecutors or lawyers may also have relevant experience and valuable insights regarding the family justice field. Although the composition of the sample, consisting solely of judges, may be seen as a limitation, given the substantial scarcity of research reporting directly on the views of family court judges dealing with PA, particularly in the Portuguese context, this research should be seen as a relevant contribution. Furthermore, this sample was geographically wide-ranging and diverse, including judges practicing in courts throughout several regions and islands of Portugal. Additionally, an equal number of males and females provides a broader enrichment of knowledge on PA issues.

The fact that the collected data were drawn from judges’ points of view and memories regarding the phenomenon, particularly in the legal context, can lead to biases regarding the descriptions and conceptualizations surrounding PA. Therefore, it is possible that some judges’ descriptions and theories may perpetuate misunderstandings about PA-related issues. However, these results should be considered as contributing to refining and developing connections between the conceptual and practical experiences in the legal context and since this is a qualitative study, it is relevant to consider that “points of view” are collected which, in essence, are only “views [perspectives] from a given point”.

As previously mentioned, it should be noted that, due to the qualitative nature of this study, it was impossible to completely eliminate the researchers’ subjectivity. The possibility for participants to read through the data and analyses and provide feedback on the researchers’ interpretations of their responses, i.e., respondent validation, could have allowed verification for inconsistencies, challenging the researchers’ assumptions, and providing them with an opportunity to re-analyze their data. However, this would require a lot of time and availability of the professionals, who are struggling with a lack of time in their daily work. Thus, in an attempt to minimize the interference of subjectivity, the basic requirements established by the grounded theory methodology were followed, namely the continuous use of reflectivity that allowed the researchers to assume a critical position towards the data [23,26]. Memo writing and ongoing discussion within the research team enabled the researchers to be aware of and acknowledge their contribution to the construction of meanings throughout the process.

The present study contributes to evidence-based knowledge about family court judges’ critical thinking on the PA concept and its manifestations in legal practice which can be relevant not only to their practices but also to the intersection between legal and psychological or psychosocial responses. A shared vision among legal professionals and between legal and psychosocial professionals is an important step towards preventing misuses and misapplications of the PA concept, thus improving more informed and comprehensive assessments, legal decision-making processes and recommendations for psychological interventions. 

### 4.4. Future Directions

This study focuses on the conceptualization of the PA phenomenon based on the views of family court judges. The authors acknowledge the relevance of continuing this line of research, searching for connections to procedures, decisions and lines of intervention of the legal system. As discussed by Warshak [43], evaluators and judges who do not consider the particularities of ABs and alienating processes in their evaluations are likely to reach false conclusions or make recommendations that are not in line with the child’s best interests. Moreover, false positive identifications of PA in family law cases may lead to concerns about its use or skepticism about the concept. In line with this concern, a manuscript on the modus operandi (including decision-making process and best practices) in the Portuguese legal system in cases of PA is currently under preparation.

Moreover, diversified samples, especially with regard to participants’ professional role, might capture complementary and interrelated views of PA. First, the involvement of other family justice practitioners—e.g., lawyers; public prosecutors—would contribute to an accurate mapping of PA. Second, the study of these processes would be enriched by other informants—e.g., social workers and psychologists; child custody evaluators; parent-child contact supervisors—, who have different roles in the intervention and access parts of family history that may be relevant to understanding PA dynamics.

Finally, it would be interesting to continue to study the phenomenon cross-culturally, particularly with the aim of determining whether legal, social or cultural factors affect the processes that nurture PA and the responses to it. More than systematizing different legal systems around the world—which has been an important focus of research (e.g., see [4,8], for review)—it would be important to explore how the phenomenon expresses itself, if it is raised as a social problem according to the contextual reality, if there are unrevealed PA patterns and other possible responses according to different countries and cultures.

## 5. Conclusions

The processes that characterize PA have repeatedly challenged professionals from different but interconnected areas to look at a complex picture of relationships, interactions and family dynamics around this phenomenon. 

This study contributes to a relevant step towards a deeper understanding of legal professionals’ views on PA. This in-depth knowledge uncovers intersections between Psychology and Law and, ultimately, promotes a comprehensive and integrated discussion on influencing factors and contexts. The authors believe that the field of Psychology has amount to offer to legal professionals, especially decision-makers. Avoiding the schism between these two fields could favor more suitable court decisions and therapeutic interventions.

## Data Availability

Not applicable.
